# Coming of Age: CD96 Emerges as Modulator of Immune Responses

**DOI:** 10.3389/fimmu.2018.01072

**Published:** 2018-05-17

**Authors:** Hristo Georgiev, Inga Ravens, Georgia Papadogianni, Günter Bernhardt

**Affiliations:** Institute of Immunology, Hannover Medical School, Hannover, Germany

**Keywords:** CD96, immunoglobulin superfamily, CD155, CD226, TIGIT, NK cells, T cells, immune regulation

## Abstract

CD96 represents a type I transmembrane glycoprotein belonging to the immunoglobulin superfamily. CD96 is expressed mainly by cells of hematopoietic origin, in particular on T and NK cells. Upon interaction with CD155 present on target cells, CD96 was found to inhibit mouse NK cells, and absence of this interaction either by blocking with antibody or knockout of CD96 showed profound beneficial effects in containment of tumors and metastatic spread in murine model systems. However, our knowledge regarding CD96 functions remains fragmentary. In this review, we will discuss structural features of CD96 and their putative impact on function as well as some unresolved issues such as a potential activation that may be conferred by human but not mouse CD96. This is of importance for translation into human cancer therapy. We will also address CD96 activities in the context of the immune regulatory network that consists of CD155, CD96, CD226, and TIGIT.

## Introduction

**Figure 1:**
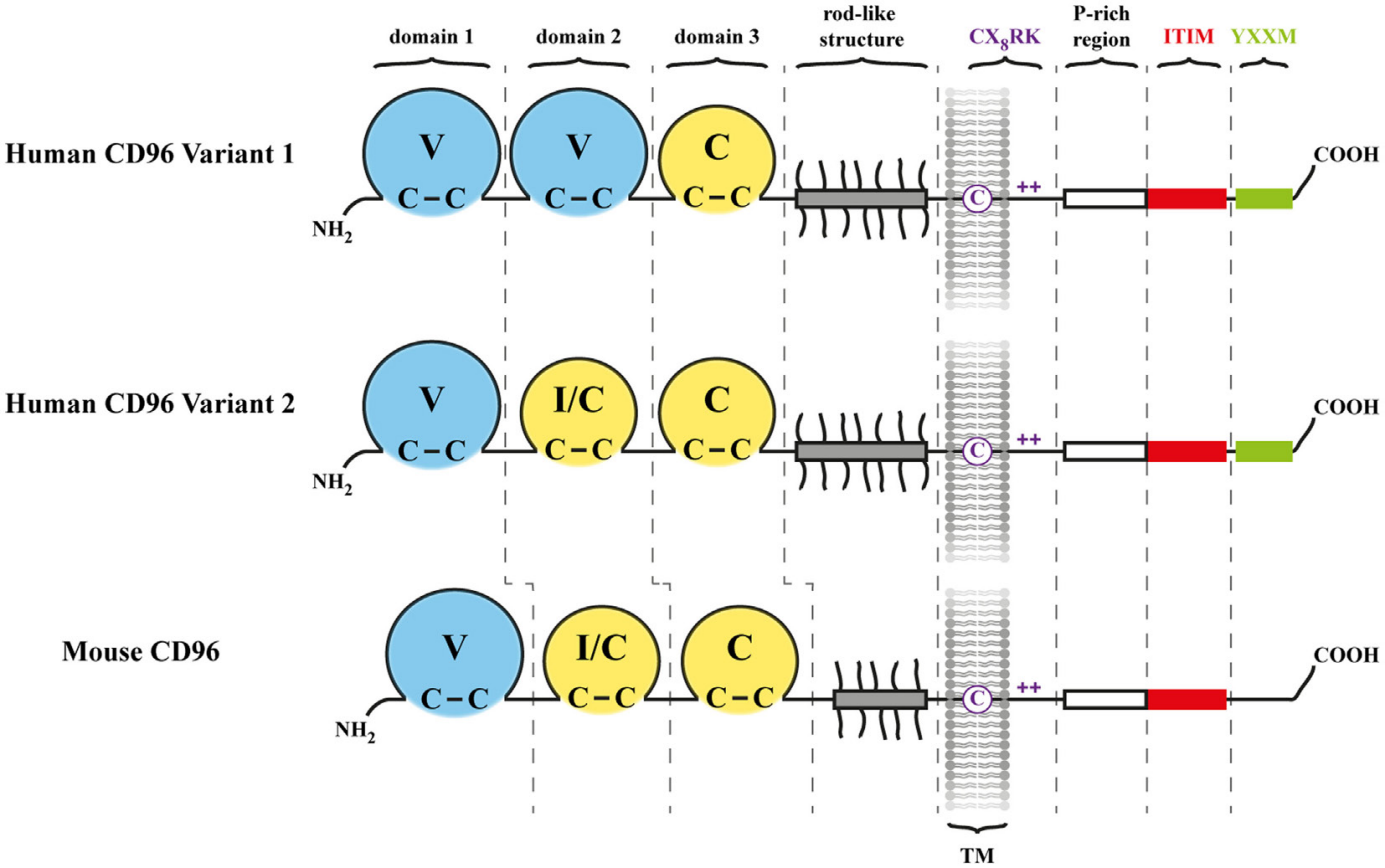
Architecture of CD96. Shown are the two human CD96 (hCD96) isoforms (variant 1 and variant 2) along with mouse CD96 (mCD96). Three Ig-like domains comprise the N-terminal (NH_2_) part of CD96 in mouse and hCD96 where V indicates a V-like domain and C indicates a C-like domain. The second domain is predicted to fold as an I-like or C-like domain in hCD96 variant 2 and mCD96. The proline/serine/threonine-rich region (gray bar) contains many potential O-linked sugar modification sites (short protrusions) and may adopt a rod-like shape. The transmembrane (TM) and cytoplasmic domain harbors motifs of potential importance for signaling triggered by CD96 as described in the text and in more detail in Figure 3. The C denotes a cysteine residing in the TM region, and the + indicates positively charged amino acid residues.

Human CD96 (hCD96) was discovered in 1992 and named originally “T cell activation, increased late expression” (1) (Figure 1). Although identified as a marker distinguishing a subset of acute leukemias (2, 3), hCD96 did not receive further attention for more than a decade. This changed when human CD155 (hCD155), formerly addressed as receptor for poliovirus (PVR), was detected as an interaction partner mediating cell adhesion (4). Furthermore, these findings suggested a role of the hCD155/hCD96 axis in target cell elimination by NK cells. Ironically, Wang et al. (1) already mentioned PVR in their publication because it showed up among other polypeptides in a similarity search. Indeed, CD96 (Figure 1) and CD155 are membrane bound receptors of the immunoglobulin superfamily (IgSF) and are distantly related to each other (5). However, in contrast to hCD155 that is expressed by a huge variety of cell types, available data indicated that hCD96 expression is largely restricted to cells of hematopoietic origin, in particular to T and NK cells (1, 4). This was confirmed by a study of mouse CD96 (mCD96) (6). Yet attempts to demonstrate a direct role of mCD96 in NK-mediated killing *in vitro* failed (6), a flaw that was resolved later on when it was shown that mCD96 can suppress NK cells *in vivo* (7). Like hCD96, hCD155 initially was an orphan receptor with no known cellular function apart from serving as the cellular receptor for PVR (8). CD155 is related to nectins (nectin 1–4) that mediate homophilic cell adhesion (9). However, in contrast to nectins, CD155 does not interact with itself in *trans*. Instead, it was reported to bind to nectin-3 assisting in the establishment of adherens junctions between tissue cells (10, 11). Moreover, CD155 is engaged in regulation of cell movement and proliferation (12–14) explaining why it was found to be a tumor antigen, first in rodents (15–17), later on also in human (18). Nowadays, hCD155 is firmly established as a marker for various types of cancer, and several reports had shown that the degree of hCD155 overexpression correlates positively with poor prognosis (19). CD96 and especially CD155 accumulated considerable sequence diversity at the amino acid level between man and mouse. Nevertheless, the interaction of CD96 with CD155 was preserved and co-evolved with species in that hCD155 only binds hCD96 but not mCD96 and *vice versa* (6, 20, 21). This corroborates the biological significance of this liaison.

**Figure 2:**
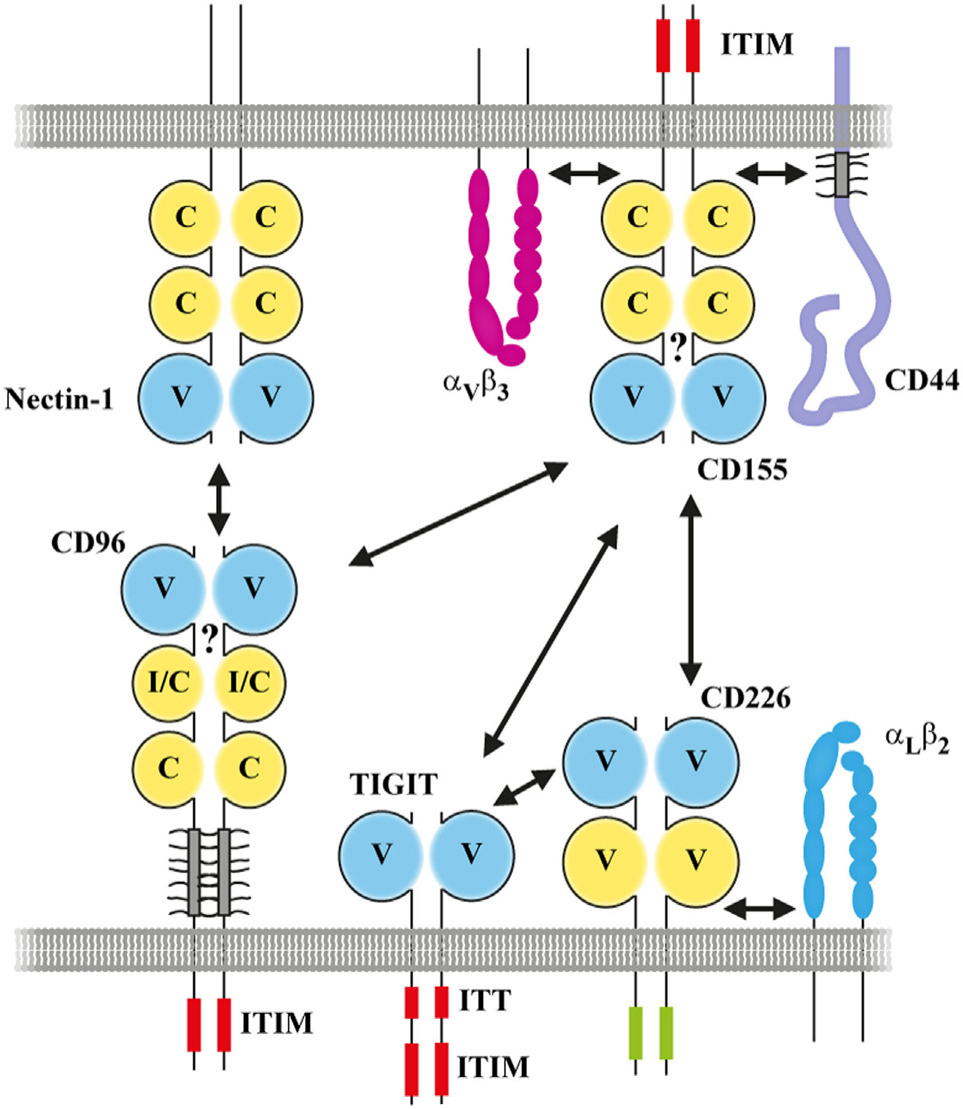
Interactions in *cis* and in *trans* providing the platform for CD96 functions in the context of the CD155 network. Interactions are indicated by two-sided arrows in black. The question mark indicates that it was not shown so far whether CD96 can form a *cis*-homodimer on the cell surface. Red boxes: ITT and ITIM, respectively. Green box: the cytoplasmic domain of CD226 containing a tyrosine and serine residue critically involved in signaling (Y_322_ and S_329_ in human). Not all interactions that can be engaged by CD155 family members are shown. Moreover, the associations shown in *cis* between CD155 family members and partner molecules are cell type specific and/or depend on a cells activation status. Please note that the molecular proportions of the given molecules are not drawn to scale to highlight the interactions between CD155 family members.

In this review, we will focus on common structural and functional aspects of CD96 that are conserved between man and mouse. But we will also highlight species-specific differences as well as gaps in our knowledge illustrating that there is still a way to go to understand comprehensively the role of this receptor in immune regulation and surveillance. By necessity, this will encompass in part a discussion of the functional context into which CD96 is embedded on the molecular level, in particular the receptors that like CD96 interact with CD155 in *trans*: CD226 (DNAM-1) (22) and TIGIT (WUCAM, VSTM3) (23). Like for CD96, binding of TIGIT (23–25) and CD226 (26, 27) to CD155 is well conserved between species. In fact, nectins, CD155, CD96, CD226, and TIGIT represent a subfamily of related IgSF receptors constituting a stimulatory/inhibitory network (Figure 2). For convenience, we will address these receptors as CD155 family members here and distinguish between human (h) and mouse (m) receptors whenever appropriate. In addition, a further branch exists consisting of nectin-like molecules (28) that will not be part of the discussion because there is no indication so far that CD96 interacts with them.

## Structure of CD96

### The IgSF-Part of the Ectodomain

**Figure 3:**
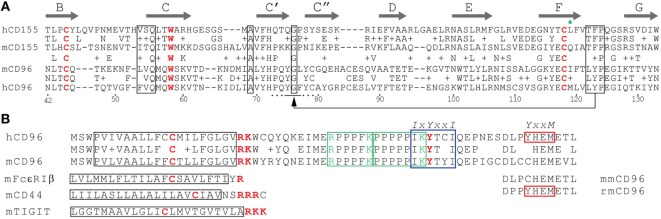
Sequence alignments of CD96 and CD155 domains. **(A)** Alignment of domain 1 of human CD155 (hCD155) and mCD155 as well as human CD96 (hCD96) and mouse CD96 (mCD96). The β-strands (thick arrows) are given according to crystal data for hCD155 (33), but the A strands were not included. A + indicates amino acids with similar chemical properties. Diagnostic residues typical of IgSF members, the cysteines forming the intra-domain disulfide bridge and tryptophan residues, are shown in red. Boxed are conserved sequences among nectins, CD155, and TIGIT that are important for homodimer and heterodimer formation as discussed in the text. The arrow highlights amino acids involved in contact formation where residues of the FG loop of one binding partner contact the C′C″ pocket of the other (located at the AX_6_G motif, A and G are boxed). The residues adjacent to the cysteine (green star) in the F strand face each other in the dimers. An additional alignment of mCD155 and mCD96 is superposed to highlight conserved amino acid residues among these distantly related receptors. **(B)** Alignment of the transmembrane (TM) and cytoplasmic domains of hCD96 and mCD96. The TM regions are boxed in black, the tandem proline-rich region in green, the ITIM motif (IXYXXI) in blue, and the YXXM motif in red, respectively. A cysteine residue located in the TM regions is highlighted in red along with the basic residues at the beginning of the cytoplasmic domain. Basic residues flanking the proline-rich motifs are marked in green and the tyrosine of the ITIM motif in red. Shown are also short corresponding amino acid sequences of mFcεRIβ, mCD44, and mTIGIT for comparison on the left side. Peptides representing these regions in mFcεRIβ, mCD44, and mCD28 bound to the SRC-related kinases LCK and LYN (34). To the right, the C-terminal CD96 residues of gray mouse lemur (*Microcebus murinus*, mmCD96) and rhesus monkey (rmCD96) are aligned to demonstrate that the YxxM motif is not conserved among non-human primates. Alignments of the domain 1 and the TM/cytoplasmic domains were done using the NCBI blastp suite applying standard settings, TM regions were predicted by the TMHMM Server v. 2.0.

CD96 represents a single pass transmembrane receptor that is heavily *N*-glycosylated (1, 6) (Figure 1). The crystal structure of the CD96 ectodomain is not resolved wherefore its folding pattern was deduced from comparisons with other IgSF members. According to this, the outermost domain represents a V-like domain in h/mCD96 and mediates binding to h/mCD155 in *trans* (20). A N-terminally located V-like domain is a common feature shared by all CD155 family members and as far as investigated, extracellular binding to themselves or other family members (but also to viruses) is invariantly restricted to this domain (blue in Figure 2). Available data from crystal structures of human/mouse nectins, CD155, and TIGIT revealed a consensus binding interface that consists of amino acids residing in the CC′C″FG region of the V-like domain (29–32). The laterally arranged CC′C″FG interfaces contact each other in an almost rectangular orientation forming the binding complex. An alignment of CD96 with its prime binding partner CD155 would suggest that most critical residues of the binding interfaces are conserved predicting that CD96 forms a “standard” dimer in *trans* with CD155 (Figure 3A). As a hallmark of these interactions amino acids of the FG loop [TFP in nectins/CD155 and (L/T)YP in CD96/CD226/TIGIT; called the key] of one binding partner come into contact with residues in the C′C″-loop area of the other (AX_6_G motif, arrow in Figure 3A, referred to as the lock) that build an acceptor pocket. In addition, residues in the F-strand next to the cysteine (green star in Figure 3A) forming the intra-domain disulfide bridge directly face each other and their compatibility impacts on the stability of the respective dimer. Also residues of the C-strand (boxed in Figure 3A) locate to the contact area. These residues and those comprising the lock are less well conserved among CD155 family members than those of the key region. The second domain of CD96 adopts an I/C-like folding pattern in mouse and man but in human, a V-like domain can be generated due to alternative splicing of the hCD96 pre-mRNA (20). Thus, in human but not in mouse two variants exist with respect to the ectodomain composition. By contrast, the third domain is a C-like domain in both hCD96 and mCD96.

### The Stalk Region

The three Ig-like domains are separated from the transmembrane (TM)-domain by an unusually long region that is rich in proline, serine and threonine (Figure 1). This allows for extensive O-linked glyco-modification that would confer to this domain a rod-like structure. As a consequence of this, the Ig-like domains should protrude from the glycocalyx layer markedly exposing them to contacting cells (1). Proline/serine/threonine-rich stalks are also present in other TM receptors like CD44 or CD8α/β. Interestingly, the degree of sialylation of the O-linked oligosaccharides on the CD8β chain impacts on co-receptor function during development of T cells in thymus (35, 36). Therefore, the stalk-like region of CD96 may play a role in orientation/presentation of the Ig-like domains representing a tool how cells could modulate the capacity of CD96 to interact with binding partners.

### The TM/Cytoplasmic Domain

The intracellular domain of h/mCD96 is rather short (45 amino acids) but possesses several interesting motifs of potential importance for CD96 function (Figure 3B). In accordance with this, there is a high degree of conservation between man and mouse in this domain (80% as compared with 54% for the ectodomain). A split motif consisting of an intra-TM cysteine and charged residues at the TM/cytoplasmic border (CX_8_RK) may serve for constitutive association with SRC-like kinases (34). Similarly composed motifs are present in other immune-relevant receptors such as CD28, CD2, CD4, CD8α, FcεRIβ, TIGIT, and CD44 (Figure 3B and not depicted). In mCD44, the intra-TM cysteine residue is of critical importance for kinase association (34). Interestingly, the very same residue that is conserved across species was shown to be crucial for homo-dimerization of hCD44 following cell activation (37, 38). Only upon covalent dimerization (not simply clustering), hCD44 can bind efficiently to its ligand hyaluronic acid and initiate signal transduction. Another feature conserved between hCD96 and mCD96 is a proline-rich (P-rich) tandem (RPPPFKPPPPPIK) that is flanked by arginine and lysine residues (Figure 3B). A similar but longer P-rich sequence was found in FasL (39). P-rich motifs represent binding sites for SH3 domain containing signaling components (40). In FasL, binding of SRC-like kinases triggers tyrosine phosphorylation and along with mono-ubiquitination of the flanking lysine residues this results of FasL sorting into secretory lysosomes (41). There is a partial overlap of the P-rich stretch with the ITIM-consensus sequence that is also conserved between man and mouse. Remarkably, Wang et al. already stressed the notion that also CD2 harbors P-rich regions in its cytoplasmic tail (1) and one of these (sequence: KGLPPLP) was shown later on to be involved in activation of integrin β1 *via* antibody mediated hCD2 cross-linking (42). This pathway requires recruitment of PI3 kinase. Although the KGLPPLP sequence does not bind to the p85 subunit of PI3 kinase, it is crucial for CD2-triggered PI3-kinase activity. In hCD96 but not mCD96, a binding of the p85 subunit *via* its SH2 domain could be accomplished by the adjacent YXXM motif that is known to bind also other signaling relevant modules in the cytoplasmic domains of CD28, ICOS-1, and CTLA-4 (43). The mutation creating the YXXM motif apparently occurred late during evolution since it is not present in all primate species (Figure 3B). Taken together, considering the tight packaging of consensus sequences for cytoplasmic binding partners, surprisingly little is known about their relevance for CD96 function.

## Dissecting CD96 Functions in Comparison with CD226 and Tigit: A Snapshot

CD96 belongs to a network of interactions that manipulates in a multifaceted fashion adhesion, activation, and inhibition of participating cells (Figure 2). CD226 was reported to activate T and NK cells (22, 44, 45) whereas TIGIT (23, 46, 47) and CD96 (7) act as inhibitors upon interaction with CD155-expressing cells. The described interaction network exists in both mouse and human. Also the functional activities triggered by its engagement appear identical to a large extent despite some black boxes. Most importantly, a direct inhibitory role of CD96 was proven only for murine NK cells and explored *in vivo* mainly in the context of tumor models (next paragraph). Conclusive evidence that this also applies to human NK cells is missing so far (48). In addition, there is currently a wealth of data documenting that CD226 activates T and NK cells but with regard to TIGIT, most publications demonstrate its role in inhibiting T cells, especially CD8 T and regulatory T cells [e.g., Ref. (49–53)]. Less data were presented that documented an inhibition of CD4 T or NK cells by TIGIT (47, 54–56). It remains to be seen whether this illustrates a functional bias of these two inhibitory receptors in that TIGIT predominantly suppresses CD8 T and regulatory T cells whereas CD96 mainly inhibits NK cells. Possibly, this view is misleading and just reflects the current lack of information especially regarding CD96 that was much less thoroughly investigated compared with CD226 or TIGIT.

## The Inhibitory Potential of CD96 Present on NK Cells

The first study characterizing hCD96 functionally implied an enhancing effect of the hCD96/hCD155 interaction on NK cell mediated cytotoxicity (4). It was demonstrated that engagement of freshly established polyclonal human NK cell lines *via* an anti-hCD96 monoclonal antibody (mAb) can promote lysis of P815 cells in a redirected killing assay. By contrast, Stanietsky et al. failed to confirm this in a similar setup. Instead, a rather mild boosting effect contributed by hCD96 on 2B4- and NKp30-mediated killing was observed (47). Importantly, attempts to demonstrate a direct role of CD96 as activator for NK cell-mediated cytotoxicity *in vitro* failed because neutralizing anti-CD96 mAb did not reveal any effect of hCD96 in killing of ovarian carcinoma cells (57) or myeloma cell lines (58) and of mCD96 in elimination of RMA, RMA-S, or YAC-1 tumor cells (6). A landmark publication addressing the function of mCD96 was published in 2014 by the group of Smyth (7). In a series of elegant experiments, Chan et al. demonstrated that mCD96 deficient (CD96^−/−^) mice were significantly more sensitive to LPS-induced endotoxicosis than wild-type (WT) mice. This was due to an increased production of IFNγ by NK cells in the CD96^−/−^ animals. Remarkably, this phenotype was not observed in TIGIT^−/−^ mice although the majority of splenic NK cells also express TIGIT (59). This implied a dominant suppressive function of mCD96 on NK cells over mTIGIT under these experimental conditions. The level of IFNγ production by NK cells controlled by mCD96 was also shown to govern the degree of protection in MCA-induced fibrosarcoma and experimental lung metastases models. In the latter, absence of mTIGIT had no impact on the metastatic burden. The same effects were observed after *in vivo* administration of a blocking anti-mCD96 mAb in WT mice (blocking refers to blocking binding to mCD155). Furthermore, protection was based entirely on an increased IFNγ production in CD96^−/−^ mice and not on enhanced NK cell mediated cytotoxicity. This was demonstrated by *in vivo* administration of a neutralizing anti-IFNγ mAb abolishing the protective effect and by a lack of difference in the killing efficiency of B16F10 cells by CD96^−/−^ or WT NK cells. These findings provided a plausible explanation why earlier attempts to verify a role of h/mCD96 in NK mediated killing *in vitro* had failed. It appears that mCD96 mainly controls the extent of cytokine production by NK cells that critically depends on an interaction with mature dendritic cells (7) while leaving direct killing tested *in vitro* untouched. *Vice versa*, h/mTIGIT may contribute to control the latter (47, 56, 60). Yet, such functional specialization is certainly not absolute and must take into account the specific immunological context as mTIGIT was shown to manipulate IFNγ production by NK cells (54, 60). In continuation of their study, Smyth’s group evaluated in more detail *in vivo* the therapeutic potential of anti-mCD96 mAb in murine tumor models (61). Blocking of mCD96 *in vivo* conveyed protective antimetastatic activity against B16F10 melanoma, 3LL lung carcinoma, LWT1 melanoma, and RM-1 prostate carcinoma cells. The antimetastatic activity of mCD96 blocking was largely abolished when mCD226 was neutralized concomitantly corroborating that an imbalance of the CD155/CD226/CD96 axis impacted on metastatic spread. The beneficial effects of mCD96 blockade were independent of antibody-dependent cell-mediated cytotoxicity (ADCC) because they continued to exist in mice lacking Fc receptors. Moreover, the combined administration of anti-CD96 mAb with anti-PD-1 mAb or anti-CTLA-4 mAb, which are therapeutically used as immune checkpoint blockade antibodies, led to significantly reduced numbers of lung metastases and increased survival in comparison with treatment with anti-PD-1 mAb or anti-CTLA-4 mAb alone. Of interest, the antimetastatic treatment was still effective though reduced in power when mAbs were given with delay. Consistent with the previous study by Chan et al. (7), the antitumor effect was mediated by an elevated IFNγ production by NK cells and an increased tissue infiltration rate but was not caused by augmented killing of target cells. This was corroborated by the finding that the antimetastatic effect of CD96 blockage was still present in perforin deficient mice but was completely abolished in the presence of neutralizing anti-IFNγ mAb. Again, TIGIT^−/−^ mice challenged with the same tumor models showed no significant reduction in numbers of tumor metastases in comparison with WT mice. Although there was no evidence proving the direct *in vivo* involvement of mTIGIT alone in controlling tumor metastases in these models, there was a synergistic effect of mCD96 and mTIGIT since blocking of mCD96 with anti-mCD96 mAb in TIGIT^−/−^ animals caused a higher degree of reduction of the numbers of tumor metastases in comparison with anti-mCD96 mAb administration in WT animals (61). The effects of an mCD96 blockade in the context of combined therapeutic approaches were refined further in a recent study utilizing pancreatic ductal adenocarcinoma (PDAC) in mice as a model for highly disseminating cancers which are largely resistant to checkpoint blockage immunotherapies (62). A set of *in vivo* experiments revealed that treatment with an anti-PD-1 mAb as a neoadjuvant in addition to chemotherapy efficiently suppressed local tumor recurrence and improved survival. Still, this approach could not effectively control distant metastases. Remarkably, an additional administration of a blocking anti-mCD96 mAb (clone 6A6) but not of a non-blocking mAb (clone 8B10) as an adjuvant following resection of the primary tumor most significantly improved the long-term survival and reduced the recurrence incidence of PDAC (62). Cytokine production was not evaluated in this study though an abrogation of the protective effect was observed following NK cell depletion. These results demonstrated the importance of a coordinated treatment regimen addressing NK and T cells for a successful therapy. Moreover, disrupting an ongoing functional interaction of mCD96 with mCD155 was crucial for NK-mediated containment of metastatic spread. However, upon transfer of B16F10 cells into mCD155-deficient recipients, the non-blocking mAb 8B10 (but not clone 6A6) retained some antimetastatic activity (63). It should be noted, though, that in this particular setting, the transferred tumor cells express mCD155 and that NK cells in mCD155 knockout hosts possess more mCD226 on their surface than NK cells in WT animals (64). Although these special parameters make an interpretation of the result by Aguilera et al. (63) difficult, it illustrates that the therapeutic effects of individual antibody clones may rely on several mechanisms to a different extent depending on the case under investigation.

## NK Cell Expressed hCD96 as Therapeutic Target in Cancer

Despite the fact that there are increasing numbers of cases documenting mCD96 involvement in controlling tumors and their metastases in mouse models, up to date there is no study translating a concept of an mAb-based neutralization of CD96 into human therapy. However, the design of such treatment strategies is impaired by the lack of conclusive evidence as to whether hCD96 inhibits or activates human NK cells. Since investigations *in vitro* were not helpful in this regard (see above), the *ex vivo* analysis of NK cells obtained from tumor patients could provide at least indirect evidence. This is exemplified by hCD226 that is frequently downregulated as part of an immune evasion mechanism in NK cells controlling tumors overexpressing hCD155 [for example, in ovarian cancer (57), for a review, see Ref. (65)]. Unfortunately, analogous information for hCD96 is very limited yet would suggest that in cases of pancreatic cancer hCD96 rather activates human NK cells (66). However, more studies are required to corroborate this.

## hCD96 in Diagnosis and Potential Therapeutic Target in Acute Myeloid Leukemia (AML)

In contrast to the role of CD96 participating in immune surveillance of tumors, hCD96 itself was identified as tumor marker. Indeed, well before first studies deciphered its functions, hCD96 was reported to be upregulated in subpopulations of T-acute lymphoblastic leukemia and AML (2, 3). Increased expression of hCD96 was shown in several subsequent studies to correlate with poor prognosis and enhanced resistance to chemotherapy [see, for example, Ref. (67, 68)] firmly establishing hCD96 as a diagnostic marker. Following the hierarchical theory of cancer development (69), it is assumed that in leukemia the disease-causing incident(s) occur among stem cells generating a leukemic stem cell (LSC) that shares self-renewal potency with the stem cells (70, 71). In line with this, Hosen et al. identified hCD96 as a potential target in an LSC-specific therapy to treat AML (72). In approximately two-thirds of the AML cases analyzed, the majority of AML-LSC was found to be hCD96^+^ whereas only a small fraction of approximately 5% was hCD96^+^ among hematopoietic stem cells from healthy donors. A promising treatment strategy would therefore be to sort out hCD96-expressing stem cells before autologous transplantation of AML patients. A classical approach of an hCD96-based therapy would engage mechanism such as ADCC and complement dependent-cytotoxicity to eliminate AML cells but must take into account that this might affect other hCD96-expressing cells as well (72–74). The functional role hCD96 plays in AML-LSC biology remains elusive, and its expression may turn out irrelevant or of inferior importance for the neoplastic properties of these cells but raises the question whether hCD96 would exert inhibition as observed for mCD96 in NK cells.

## Function of CD96 in T Cells

Although identified originally as a human T cell antigen (1), not much is known about CD96 function in CD4 and CD8 T cells. Recently, the level of hCD96 expression on CD8 T cells from HIV-1-infected patients with high and low viral loads was analyzed (75). Interestingly, a dowregulation of hCD96 on a fraction of CD8 T cells present in the patients with high viral loads was found. Functional characterization of the hCD96^+^ and hCD96^−^ CD8 T cells showed that both are potent producers of IFNγ but that the hCD96^−^ cells also produced perforin. This raises the possibility that in chronic infection hCD96 negatively regulates perforin production in human CD8 T cells. Dissimilar effector functions were also observed among mCD96^hi^ and mCD96^lo^ TH9 cells generated *in vitro* (76). The mCD96^hi^ subpopulation was found to be less pathogenic, produced less cytokines, and propagated less efficiently when compared with mCD96^lo^ TH9 cells. These observations would be in line with the assumption that CD96 inhibits selective T cell effector functions. But again, more information is required to draw more general conclusions.

## Unresolved Issues, Future Challenges

### Interaction Partners of CD96 in *Cis*

Despite the existence of various consensus binding sites, the nature of the *cytoplasmic* interaction partners binding to CD96 remains a subject of speculation. The elucidation of the signaling pathways triggered upon CD96 engagement will be crucial for a better understanding of the CD96 biology. But functions of CD96 may also be regulated by *extracellular* proteins complexing *in cis* thereby creating more or less heterogeneous membrane complexes. The most simple higher order structure would be a homo-dimeric CD96 receptor. To manipulate the monomer/dimer balance represents a well-known tool how cells can control the functional status of receptors that depend on *cis*-dimerization (e.g., CD44 as discussed earlier). Experimental evidence would suggest that dimerization of CD155 in *cis* is required for functionality (11, 28) and *cis*-dimerization appears to be a common theme for CD155 family members. Interestingly, a high molecular weight complex (~240 kDa) in addition to the presumptive monomeric hCD96 (~160 kDa) was described by Wang et al. (1) investigating hCD96 by SDS-PAGE analysis under non-reducing conditions following immunoprecipitation. However, the precipitated material obtained from the human T cell lines migrated too fast for a hypothetical homo-dimer (~320 kDa) raising doubts regarding its composition. Thus, it remains unclear whether membrane-bound CD96 forms dimers in *cis* and whether this is required for functionality. As described, CD155 family members possess a binding interface in domain one that is used for complex formation with other members in *trans*. The very same CC′C″FG interface can be utilized by nectins and most likely also by CD226 to form homo-dimers in *cis*. However, in contrast to nectins, available data suggest that the CC′C″FG interface of CD155 is ineligible to perform homo-dimerization (11, 30). This fits the observation that, unlike nectins, CD155 does not mediate homophilic cell adhesion. Although not proven, it is plausible to assume that this characteristic is also shared by CD96 that like CD155 lacks self-adhesive capacity (6). Therefore, any potential *cis*-dimerization must utilize alternative mechanisms to accomplish this such as the TM cysteine (Figure 3B) that may serve to form stable CD96 dimers. Its genetically engineered replacement by another residue might inform whether the high molecular weight component observed by Wang et al. represented indeed a dimer (1) or whether another component stably associated with hCD96. The integration of CD96 into a hetero-dimeric/-oligomeric structure on a cell surface is quite likely considering other CD155 family members. CD155 was found to be associated with the integrin α_ν_β_3_ in fibroblasts (11, 77) or hCD44 on monocytes (78) (Figure 2) and CD226 complexes to LFA-1 in NK and activated T cells (79, 80). Integrin association in *cis* with CD155 (11, 81) or CD226 (80, 82, 83) is of functional relevance. Fuchs et al. (4) reported that in their redirected killing assays using an anti-hCD96 mAb activated polyclonal NK cells but not the cell line NK92 was stimulated to kill target cells. This illustrates that hCD96 expressed by NK92 cell differs functionally from that of the freshly isolated NK cells (4). Bearing in mind that hCD226 requires co-activity of β_2_-integrin for NK cell function (79), the authors speculated that a similar mode of regulation might also apply for hCD96. Integrins of the β1 family might represent candidates taking this role. The incorporation of CD96 into complex membrane-bound structures could be specific for the type of cell or its activation status (like in case of CD226). This would enable a context-dependent tuning of CD96 functions. In addition, this might also force the receptors to preferentially engage in interactions in *trans* and help avoid that, for example, CD155 and CD96 neutralize each other in *cis* since both are usually present simultaneously on the surface of T and NK cells.

### Functional Differences Between hCD96 and mCD96

An important issue that directly would affect the translation of results obtained in mouse into therapeutic approaches for treatment of diseases in human relates to the structural differences between mCD96 and hCD96 and the resulting potential functional divergences. Of note, mCD96 but not hCD96 binds to nectin-1. Overexpression of nectin-1 in tumor cells is not described, but nectin-1 serves as an entry receptor for herpesviruses in human and mouse (84, 85) and therefore control of infection *via* CD96 expressed by NK cells may differ between species. It is surprising that human nectin-1 (hnectin-1) does not bind to hCD96 because mnectin-1 and hnectin-1 are highly conserved possessing an identical CC′C″FG interface in their domain one. mnectin-1 also binds to the first domain of mCD96 wherefore it is likely that subtle differences in the CC′C″FG interface of mCD96 compared with hCD96 (Figure 3A) account for the divergent binding specificity. Also effects from outside the binding interface can contribute substantially to modulate or alter binding of CD96 to ligands and thus illustrate the complexity of the CC′C″FG interface in mediating binding. The second domain of hCD96 (but not mCD96) can adopt a V-like folding pattern due to alternative splicing, and the presence of this domain instead of the I/C-like second domain modulates binding strength to hCD155 (20). The functional significance of the two existing variants in human compared with mouse remains elusive. But quantitative PCR data would indicate that the I/C-like domain variant that binds stronger to hCD155 and that corresponds to the domain one present in mCD96 is predominantly expressed in all normal cells and tissues tested (20). Also a described point mutation in the most distant third domain of hCD96 that was linked to a rare form of trigonocephaly weakens the binding to hCD155 (20). Along with other results, this suggested that the first domain of hCD96 but not of mCD96 is quite susceptible in its binding characteristics to even remotely located anomalies. This also increases the likelihood that a modified rigidity of the stalk region due to altered glyco-modification as mentioned earlier modulates ligand binding. Last not least, reminiscent of the scenario for h/mCD96 itself, the hCD96 interaction partner hCD155 can be expressed in four different isoforms due to alternative splicing (86) whereas alternative mRNA splice variants for mCD155 were not observed (21). Two hCD155 isoforms represent secreted receptors lacking the TM domain and of the two membrane-bound versions only the α-isoform (that corresponds to mCD155) harbors an ITIM motif (Figure 2) (87). Thus, human but not murine cells expressing CD155 could create a balance between an hCD155 isoform serving as an adhesion and signaling receptor and another one that only mediates adhesion.

A critical point that awaits elucidation relates to the issue whether hCD96 possesses an inhibitory potential as revealed for mCD96 (7). The key to this is buried in the short cytoplasmic domains. Despite a high degree of conservation they differ in the absence/presence of the YXXM motif. The importance of this binding site for actual performance of hCD96 cannot be predicted due to its low degree of specificity. Thus, although both can recruit p85 of PI3 kinase, the YXXM in CD28 triggers IL-2 production upon tyrosine phosphorylation but YXXM in ICOS-1 fails to do so because GRB2 cannot be bound (88). Taken together, there might be a “worst case” scenario, and hCD96 exerts inhibition or activation depending on the cell type.

## Concluding Remarks

The regulatory network built by the CD155-family members attracted increasing attention during the past decade. However, despite its early identification, CD96 represents the least well-investigated building block of this system. Considering the importance of the CD155-driven regulatory circuits in immune surveillance in general and in particular in tumor biology, it is of upmost interest to learn more about the pathways governing the functions of hCD96. Current evidence brings to mind that the CD155 network is rather complex, and many factors contribute to the net inhibitory/activating outcome of its engagement (7, 19, 59, 89–91): participating cell types, divergent affinities of the receptors among each other, splice variants, the variegated expression dynamics that change with cell status, the accessory molecules that may associate with family members in a cell type- and status-specific pattern. This listing is certainly incomplete. This illustrates that the biological significance of CD96 can only be apprehended adequately when studied as part of this network.

## Author Contributions

HG and GB designed the concept and wrote the manuscript. IR and GP contributed to the overall concept of the manuscript, helped designing figures, and assisted in editing the manuscript.

## Conflict of Interest Statement

The authors declare that the research was conducted in the absence of any commercial or financial relationships that could be construed as a potential conflict of interest.
